# Universal scaling of extinction time in stochastic evolutionary dynamics

**DOI:** 10.1038/s41598-022-27102-0

**Published:** 2022-12-27

**Authors:** Ching-I Huang, Chun-Chung Chen, Hsiu-Hau Lin

**Affiliations:** 1 Department of Physics, National Tsing Hua University, Hsinchu, 30013 Taiwan; 2 Mathematics Institute, University of Warwick, Coventry, UK; 3 Zeeman Institute for Systems Biology and Infectious Disease Epidemiology Research, University of Warwick, Coventry, UK; 4 Institute of Neuroscience, National Yang Ming Chiao Tung University, Taipei, 112304 Taiwan; 5 Institute of Physics, Academia Sinica, Nankang, Taipei, 115201 Taiwan

**Keywords:** Evolutionary theory, Physics, Statistical physics, thermodynamics and nonlinear dynamics

## Abstract

Evolutionary dynamics is well captured by the replicator equations when the population is infinite and well-mixed. However, the extinction dynamics is modified with finite and structured populations. Experiments on the non-transitive ecosystem containing three populations of bacteria found that the ecological stability sensitively depends on the spatial structure of the populations. Based on the Reference–Gamble–Birth algorithm, we use agent-based Monte Carlo simulations to investigate the extinction dynamics in the rock-paper-scissors ecosystem with finite and structured populations. On the fully-connected network, the extinction time in stable and unstable regimes falls into two universal functions when plotted with the rescaled variables. On the two dimensional grid, the spatial structure changes the transition boundary between stable and unstable regimes but doesn’t change its extinction trend. The finding of universal scaling in extinction dynamics is unexpected, and may provide a powerful method to classify different evolutionary dynamics into universal classes.

## Introduction

The paramount question in evolution is how to maintain robust biodiversity in an ecosystem hosting mutually competing species^1–5^. The opposite side of the same question is about extinction mechanism. Derived from the evolutionary game theory, replicator equations^6–11^ deliver a deterministic description for population dynamics with frequency-dependent selection. The competitions between individuals of different species are described by the payoff matrix^12,13^ in the game theory and the resultant replicative dynamics has been rather successful in many ecosystems^11^. Despite of the general applicability with success, it is important to keep in mind that the replicative dynamics is limited to the well-mixed configurations in the infinite population limit.

What happens if the populations are finite and/or competitions between individuals occur on a network with specific spatial structure? Many studies^14–31^ show that ecosystems with finite populations and/or on structured networks reveal significant deviations from the replicative dynamics. For instance, in a recent study^32^ on the non-transitive rock-paper-scissors ecosystem containing three populations of Escherichia coli. They found that diversity is rapidly lost in the ecosystem when dispersal and interaction occur over relatively large spatial scales, while all species coexist when ecological competitions are localized. That is to say, the ecological stability seems to be weakened in the well-mixed populations. Their findings inspire us to explore the role of spatial structure in evolutionary dynamics.

In addition, there has been controversy over the effect of finite population in the rock-paper-scissors ecosystem. Some study^33^ shows that a critical population size $N_c$ exists: For $N>N_c$, the infinite-population behavior is recovered, while for $N<N_c$, the stochastic fluctuations arisen from finite population drives the ecosystem toward extinction. On the other hand, the other study^34^claims that the extinction time is always *finite* and the ecological stability needs to be defined by the trend of the extinction dynamics. The ecological stability of an ecosystem with finite population means that the extinction time grows exponentially with the population size, $T_{ex} \sim \exp (N/N^*)$, where $N^*$ is some characteristic constant depending on the model details. This important conflict together with the previous experimental observations encourages us to investigate these issues by numerical simulations.

To put the discussions on firm ground, we concentrate on the non-transitive rock-paper-scissors game^35–37,37–56^, known as a paradigm to illustrate the species biodiversity. Recent empirical investigations have identified several ecological systems as the rock-paper-scissors system including the coral reef invertebrates^57^, the mating strategies of lizards in California^58^, the bacterial game in vitro and in vivo^32,59,60^, the vertebrate community in the high-Arctic tundra in Greenland^61^ and the plant communities^62,63^. These findings in nature make rock-paper-scissors game not only a theoretically interesting model but also a practical system to study.

In this Report, we use agent-based Monte Carlo simulations to investigate the extinction dynamics in the rock-paper-scissors ecosystem with finite and structured populations. Making use of numerical simulations, one can extract the extinction time in different regimes. However, for the finite and structured populations, the usual Moran algorithm^64,65^ and its generalized versions^24,66,67^ may not be appropriate. One shall turn to the evolutionary theory on graphs^21,68–70^ to include the local mutual competitions. Following the same spirit, we introduce a local three-party Reference–Gamble–Birth (RGB) algorithm and perform the agent-based Monte Carlo simulations for the evolutionary dynamics. The RGB algorithm only requires local information in the stochastic processes and captures the network structure into account. And, it correctly reproduces the replicator dynamics when the populations are infinite and well-mixed.

Two major results are found in our numerical simulations. The first one is the universal scaling: the extinction time is always finite and falls onto the universal function when plotted with the rescaled variables. On the fully-connected network, two universal functions for stable and unstable regimes are found and the phase transition between them corresponds to neutral (critical) dynamics where the extinction time shows power-law behavior. The unexpected universal scaling may provide a powerful method to classify ecosystems with different dynamical behaviors into universal classes. The second major result is the notion of global payoff versus local payoff. On the two dimensional grid, the spatial structure changes the transition boundary between stable and unstable regimes but the universal function remains the same. This observation motivates us to introduce the notion of global payoff which turns out to be consistent with our numerical simulations. Our numerical simulations not only resolve the conflicting results in the previous studies^33,34^ but also suggest a potential tool to classify different evolutionary dynamics by universal scaling.

## Methods

### Rock-paper-scissors game

 Here we consider the symmetric cyclic competitions between species *A*, *B* and *C* described by the payoff matrix

1 $$\begin{aligned} P= \left( \begin{array}{ccc} 0 &{}\quad -1 &{}\quad g \\ g &{}\quad 0 &{}\quad -1 \\ -1 &{}\quad g &{}\quad 0 \end{array}\right) , \end{aligned}$$

where the positive-definite parameter *g* denotes the gain in the cyclic competitions. The replicative dynamics of the ecosystem with populations $N_A$, $N_B$, $N_C$ for the species *A*, *B*, *C* is captured by the following differential equations,

2 $$\begin{aligned} {\dot{a}}&= (gc-b)a - \phi a, \end{aligned}$$

3 $$\begin{aligned} {\dot{b}}&= (ga-c)b - \phi b, \end{aligned}$$

4 $$\begin{aligned} {\dot{c}}&= (gb-a)c - \phi c, \end{aligned}$$

where $a(t) \equiv N_A/N$ is the frequency (relative population) of species *A* in the total population $N=N_A+N_B+N_C$. The other frequencies $b(t) \equiv N_B/N$ and $c(t) \equiv N_C/N$ are defined in similar fashion. The parameter $\phi = (g-1)(ab+bc+ca)$ is the average fitness of the ecosystem.

The symmetric replicator equations^11,34^ host a non-trivial fixed point $(a^*, b^*, c^*) = (\frac{1}{3}, \frac{1}{3}, \frac{1}{3})$. If the gain is greater than unity, $g>1$, the flows around the fixed point is stable. On the other hand, if the gain is less than unity, $g<1$, the fixed point becomes unstable. At the critical gain $g_c = 1$, it corresponds to the zero-sum game and the flow around the fixed point is neutral. Therefore, according to the replicator equations, the ecosystem goes through a phase transition from the stable regime to the unstable one when the gain *g* is tuned through the critical point $g_c=1$.

### RGB algorithm

**Figure 1:**
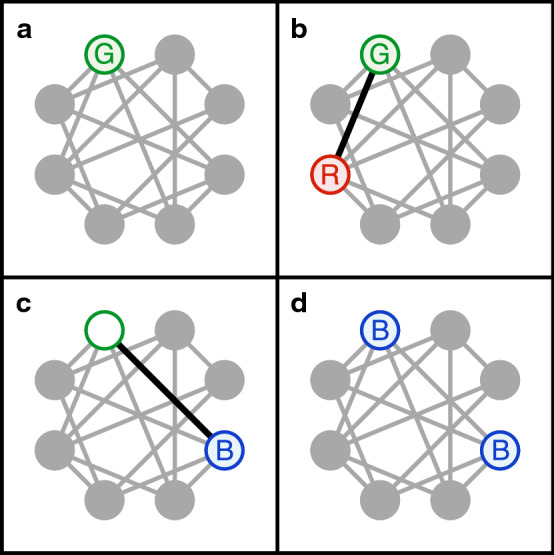
Reference–Gamble–Birth (RGB) algorithm. Individuals are represented by filled grey circles and the network for mutual competitions is shown as grey lines. For each RGB update, it consists four steps as shown here. At first, a Gamble (G) is randomly picked from the entire populations. Next, an Reference (R), randomly chosen from the neighborhood, interacts with the Gamble and sets the bar for Gamble’s fate. The death probability of Gamble ($D_{GR}$) in a single update, defined by the death matrix *D*, is solely determined by the species of Gamble and Reference. In the case of Gamble decease, a randomly chosen Birth (B) in the neighborhood reproduces and fills up the empty spot. The participating individuals of Reference, Gamble and Birth are highlighted by red, green and blue colors respectively and interact via the highlighted black links.

 The above scenario is well known for the rock-paper-scissors game when the population is infinite and well-mixed. But, what happens when the population is finite and the competitions among different species occur on the network with specific spatial structure? To investigate these important issues, we utilized the agent-based Monte Carlo simulations. To incorporate the locality of mutual competitions on the reasonable ground, we introduce a local three-party Reference–Gamble–Birth (RGB) algorithm. For each RGB update, it consists four steps as shown in Fig. 1. In the RGB algorithm, the third-party Reference provides local survival standard and determines the death probability of Gamble.

The death matrix *D* plays a central role in RGB algorithm. Similar to payoff matrix, its matrix elements $D_{ij}$ defining the death probability for all possible competitions among species. The definition of death matrix indicates that the death matrix share the same matrix dimension with the payoff matrix and all its matrix elements must sit inside the range $0\le D_{ij}\le 1$. In this section, we are going to show (i) the equivalent dynamics between replicator equations and the deterministic dynamics of stochastic updates in RGB algorithm in well-mixed and infinite populations; (ii) a simple scaling relation, i.e. $D=P/s$, to construct the death matrix *D* for RGB algorithm from an arbitrary negative payoff matrix *P*; (iii) extracting the extinction time (i.e. a simple scaling $T_{ex}=T_{RGB}/s$) for a payoff matrix *P* by using the RGB algorithm.

In a single update, there are two possible ways to change the population size $N_i$ of species *i*: (1) $\Delta N_{i}=1$ with probability $D_{kj}x_{i}x_{j}x_{k}$ coming from the process of the decease of a species-*k* Gamble when contacting with a Reference of species *j* and the random replacement of a Birth of species *i*, where $k \ne i$. The death probability is $D_{kj}$ in this case. (2) $\Delta N_{i}=-1$ with probability $D_{ij}x_{i}x_{j}x_{k}$ coming from the process that a species-*i* Gamble dies during the interaction between a species-*j* Reference and a Birth of species-*k* is randomly chosen to fill the empty site, where $k \ne i$. The death probability is $D_{ij}$ here. Weighting all possible competitions with the corresponding probabilities, the average change in populations after a single update takes the following form,

5 $$\begin{aligned} \frac{\Delta N_{i}}{\Delta \tau }= & {} \sum _{k \ne i}\sum _{j}D_{kj}x_{i}x_{j}x_{k}-\sum _{k \ne i}\sum _{j}D_{ij}x_{i}x_{j}x_{k}\nonumber \\= & {} \sum _{j,k}\left( D_{kj}-D_{ij}\right) x_{i}x_{j}x_{k}, \end{aligned}$$

where $\Delta \tau$ is the virtual time interval in the numerical simulation. However, within a realistic time interval $\Delta t$, we expect the number of encountering events is proportional to the population size *N*^24^, i.e. $\Delta \tau = N \Delta t$. Thus, the real time dynamics in the continuous limit when the population becomes infinite is

6 $$\begin{aligned} \frac{dx_{i}}{dt} = \sum _{j,k}\left( D_{kj}-D_{ij}\right) x_{i}x_{j}x_{k}, \end{aligned}$$

where $x_{i}=N_{i}/N$ denotes the frequency of species *i*. Defining $d_{i}=\sum _{j}D_{ij}x_{j}$ as the frequency-dependent death probability of species-*i* and $\phi _{d}=\sum _{i}d_{i}x_{i}$ as the average death probability of the entire populations, the real time deterministic RGB dynamics becomes

7 $$\begin{aligned} \frac{dx_{i}}{dt} = -d_{i}x_{i}+\phi _{d}x_{i}. \end{aligned}$$

This is the death version of replicator equation with $b_{i}=0$ for all species. The RGB algorithm introduces selection among species by a death matrix, and therefore its deterministic dynamics is captured by the replicator equations without the birth terms.

Now, we can derive the relation between the death matrix in RGB algorithm and the payoff matrix in replicator equations when frequency-dependent selection is taken into account. The gauge redundancy of the payoff matrix indicates the replicator equations remain invariant when adding a arbitrary constant to all elements in the payoff matrix^11^. Based on the gauge redundancy, we consider a negative payoff matrix *P* with negative values for all matrix elements (i.e. $P_{ij}\le 0$ for all *i* and *j*) without loss of generality. Because $0\le D_{ij}\le 1$, a proper rescaling factor is required to establish the equivalence between the payoff matrix $P_{ij}$ and the death matrix $D_{ij}$. Introducing a proper rescaling factor *s*, which has a lower bound $s_m = \text {max}(|P_{ij}|)$ defined by the payoff matrix, we define the death matrix from the negative payoff matrix as

8 $$\begin{aligned} D_{ij} \equiv - P_{ij}/s. \end{aligned}$$

In the case of frequency-dependent selection, $f_{i}=\sum _{j}P_{ij}x_{j}$, we can rewrite the frequency-dependent death probability as $d_{i}=-\sum _{j}P_{ij}x_{j}/s=-f_{i}/s$ and the average death probability of the entire populations as $\phi _{d}=-\sum _{i}f_{i}x_{i}/s=-\phi /s$. Thus, the deterministic dynamics of RGB algorithm then is related to the replicator equations by the rescaling factor *s*,

9 $$\begin{aligned} \frac{dx_{i}}{dt} = \left( f_{i}-\phi \right) x_{i}/s. \end{aligned}$$

As shown in the above equation, the RGB algorithm generates a rescaled population dynamics of replicator equations with the rescaled fitness $f_{i}/s$. The rescaled fitness in RGB algorithm reduces the strength of competition and therefore slows down the dynamics of replicator equations by a factor of *s*. Thus, the extinction time obtained by the numerical simulations should also be rescaled by the same factor, i.e. $T_{ex}=T_{RGB}/s$, accordingly to yield the extinction time of replicator equations.

The RGB algorithm has several advantages over the Moran process. First of all, only local information is involved in the stochastic process, more realistic than the need of global information for the Moran process. Secondly, the RGB algorithm reproduces the dynamics of frequency-dependent selection described by the replicator equations in infinite and well-mixed populations when the rescaling of time is restored properly. Finally, the the RGB algorithm takes the network structure into account, capturing the sensitive dependence of evolutionary dynamics in structured populations.

## Results

### Universal scaling in well-mixed populations

**Figure 2:**
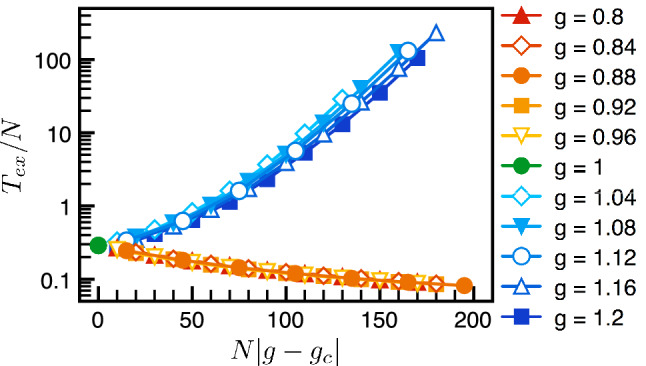
Scaling functions of extinction time in well-mixed populations. Numerical results show three types of scaling forms (exponential, logarithm and linear) depending on the values of the local gain *g*. Plotted versus the scaling variable $x \equiv N |g-g_{c}|$, the rescaled extinction time $T_{ex}/N$ falls onto two distinct scaling function $F_{\pm }(x)$. The stable and unstable regimes are represented by the blue and red color systems while the critical point is represented by the green color.

 We perform agent-based Monte Carlo simulations to extract the mean extinction time. For each run, when one of the species goes extinct, we stop the simulated evolution and mark the time of extinction. The mean extinction time $T_{ex}(N,g)$, depending on the population size *N* and the gain *g* defined in the payoff matrix, is computed by taking ensemble average of 10,000 runs in numerical simulations. We first focus on the well-mixed population, i.e. the fully connected network where every individual is connected to each other. It is rather remarkable that all numerical data can be collapsed onto the universal functions,

10 $$\begin{aligned} T_{ex}(N, g) = N^\alpha F_{\pm } \left( N^{\beta } \Delta g \right) , \end{aligned}$$

where $\Delta g \equiv |g-g_c|$ denotes the deviation from the critical gain $g_c=1$. As shown in Fig. 2, the numerical data for $g>g_c$ (stable regime) and $g<g_c$ (unstable regime) collapse onto the universal scaling functions $F_{\pm }(x)$ respectively. The exponent $\alpha =1$ can be extracted from extinction time of different population sizes at the critical point $\Delta g =0$ and the other exponent $\beta =1$ is the optimal fitting to collapse all data with different *N* and *g*. In the following paragraphs, we would like to show that these scaling variables can be understood within the framework of the renormalization group (RG)^71^. But, it is worth mentioning that the scaling variable $x = N(g-1)$ agrees with the our expectation from population genetics^72^ that the extinction time in finite populations will scale as the product of population size and selection strength.

Let us derive the scaling form of the extinction time^71^ by RG analysis. Assuming the scaling dimensions for $T_{ex}$, *N* and $\Delta g$ are $d_T$, $d_N$ and $d_g$ respectively,

11 $$\begin{aligned} {\widetilde{T}}_{ex}({\widetilde{N}}, \Delta {\widetilde{g}} )= & {} b^{d_T} T_{ex}(N, \Delta g), \end{aligned}$$

12 $$\begin{aligned} {\widetilde{N}}= & {} b^{d_N}N, \end{aligned}$$

13 $$\begin{aligned} \Delta {\widetilde{g}}= & {} b^{d_g} \Delta g. \end{aligned}$$

Thus, the extinction time takes the following scaling form,

14 $$\begin{aligned} {\widetilde{T}}_{ex}(b^{d_N}N, b^{d_g} \Delta g) = b^{d_T} T_{ex}(N, \Delta g). \end{aligned}$$

Choose the rescaling factor properly so that $b^{d_N} N=N_0$, where $N_0$ is some large number. The extinction time can be rewritten in terms of the rescaled variables,

15 $$\begin{aligned} T_{ex}(N, \Delta g)&= b^{-d_T} {\widetilde{T}}_{ex}(b^{d_N} N, b^{d_g} \Delta g) \nonumber \\&= (N/N_0)^{d_T/d_N} {\widetilde{T}}_{ex}[N_0, \Delta g (N/N_0)^{-d_g/d_N}]. \end{aligned}$$

Introducing two exponents $\alpha \equiv d_T/d_N$ and $\beta \equiv -d_g/d_N$, the above scaling relation can be cast in the elegant result in Eq. (10). Note that the scaling functions $F_{\pm }(x)$ for stable and unstable regimes are different but the exponents $\alpha$ and $\beta$ are universal in the vicinity of the critical point.

The emergent universal scaling is highly non-trivial. For instance, extinction time of different population sizes at the critical point all collapse onto just one point (green dot in Fig. 2). Our numerical simulations show that the extinction time is always finite but the ecological stability can still be defined through the trend of extinction time. Plotted with the rescaled variables, stable and unstable regimes fall onto two universal functions $F_{\pm }(x)$ and can be differentiated without ambiguity. In general, the exact forms of the scaling functions $F_{\pm }(x)$ are difficult to obtain. However, its asymptotic form can be understood by the following arguments. Consider the stable regime first. In the asymptotic limit ($|x|= N |\Delta g| \gg 1$), the extinction of the ecosystem arises from a chain of successive rare event against the flow back to the stable equilibrium. In the stable regime, the clusters within the correlation length are locally stable with a small probability *p* to go extinct. Suppose the typical size of the cluster is $N_c$ so that there are roughly $N/N_c$ clusters. The probability for the whole ecosystem to go extinct is thus $p^{N/N_c} = q^N$, where $q = p^{1/N_c}$. In consequence, the extinction time grows exponentially with the population size *N* and the asymptotic form of $F_{+}(x)$ should be

16 $$\begin{aligned} F_{+}(x) \sim e^{A x}&\quad \rightarrow \quad T_{ex} \sim N e^{N/N_+}. \end{aligned}$$

Here *A* is some positive constant and $N_{+} = 1/(A \Delta g)$ is the characteristic constant for the exponential growth. In the unstable regime, the population decays exponentially, $N(t)=Ne^{-\gamma t}$. The extinction time is usually estimated by the criterion $N(T_{ex} ) \approx {{\mathcal {O}}}(1)$, leading to the logarithmic trend $T_{ex} \sim \ln N$. The asymptotic form of the scaling function $F_{-}(x)$ thus takes the form,

17 $$\begin{aligned} F_{-}(x) \sim \frac{\ln x}{x}&\quad \rightarrow \quad T_{ex} \sim \ln N. \end{aligned}$$

The asymptotic forms in Eqs. 16, 17 has been derived in the previous studies^34^ and agree with the numerical simulations presented in Fig. 2 rather well. Even though the asymptotic forms of the scaling functions can be understood rather well, the exact forms of the scaling functions $F_{\pm }(x)$ are difficult to obtain.

### Global payoff matrix in structured populations

**Figure 3:**
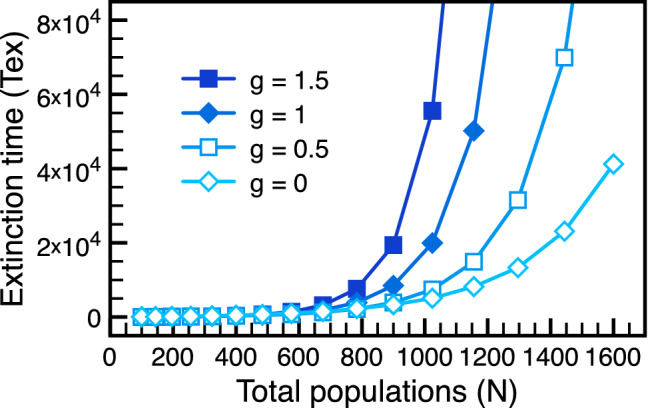
Enhanced ecological stability on 2D grid. Differnt from the phase transition observed in the fully-connected network (well-mixed population), the extinction time on the 2D grid always follows the exponential trend even for $g \leqslant 1$ where the average local payoff $g-1$ is negative. The spatial structure gives rise to non-trivial normalization on the local payoff matrix and enhances the biodiversity on the 2D grid.

 Now we turn to the extinction dynamics on the 2D grid. As explained in the previous paragraphs, only local neighbors participate in each RGB updates. It is striking that the biodiversity is enhanced due to the spatial structure of the network: the extinction time always follows the exponential trend as shown in Fig. 3. Even in the extreme case $g=0$, the stability of the cyclic-competing ecosystem is still protected by the grid structure. Is the extinction time described by the same scaling function as in the well-mixed case? The answer, after finding appropriate rescaled variable, turns out to be positive.

**Figure 4:**
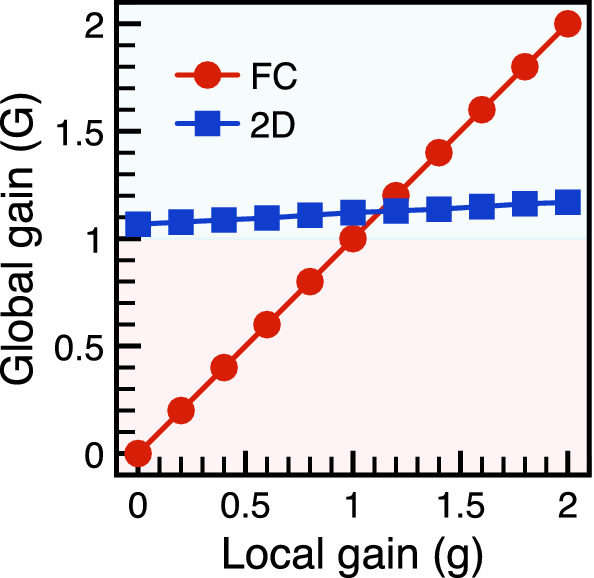
Global payoff on the 2D grid. In spatial games, the local evolutionary dynamics is generated by the local payoff matrix. However, the global ecological stability is not directly reflected by the local payoff matrix due to the renormalization from the network structure. Referencing with the fully-connected network, the global gain *G* can be extracted by equating the characteristic constant of the extinction time. The mapping between the local gain *g* and the global gain *G* not only highlights the renormalization of the payoff matrix but also provides clear evidence for robust biodiversity ($G>1$) in 2D grid.

**Figure 5:**
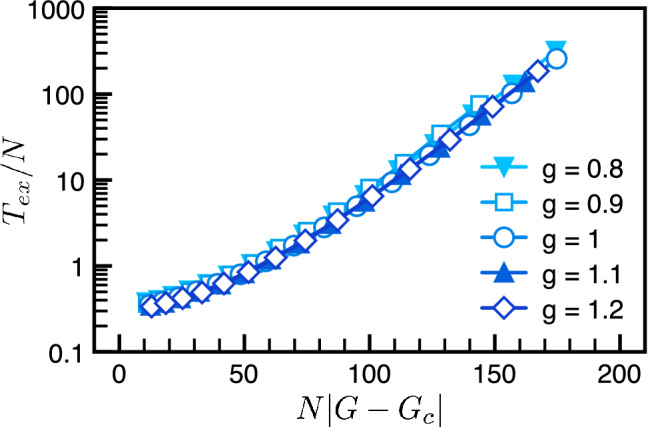
Universal scaling with the global gain. Choosing the scaling variable $x = N |G-G_c|$, the data for the extinction time in the 2D grid collapse onto the same scaling function as found in the fully-connected network. For comparison, one observes the universal function (the blue color system) in Fig. 2 is the same as that presented here.

Even though the phase boundary between stable and unstable regimes are modified by the spatial structure of the network, the universal functions remain the same. We are thus inspired to introduce the notion of global payoff matrix as explained below. Local population dynamics generated by local interactions depends on the payoff matrix parametrized by the local gain *g*. But, the extinction time is a global property: even with the same local gain *g*, the 2D grid and the fully-connected network (well-mixed population) give different results. Because the fully-connected network looks the same either locally or globally, it is invariant under renormalization-group transformation and serves as a good reference. In RG analysis, the partition function is kept invariant to extract the renormalized coupling constants^71^. Here we adopt the same method and introduce the global gain *G* on an arbitrary network via the relation

18 $$\begin{aligned} \lim _{N\rightarrow \infty }T_{ex}(N, g) = \lim _{N\rightarrow \infty } T_{ex}^{\textrm{FC}} (N, G), \end{aligned}$$

where the superscript FC stands for “fully-connected”. That is to say, the extinction time for the local gain *g* on the considered network is set equal to that for the global gain *G* on the fully-connected network. According to the definition, $g=G$ on the fully-connected network, reflecting the fact that local and global properties are the same in the well-mixed population. In our numerical simulations, the asymptotic limit is approximated by the largest population size in available simulations. To accurate the mapping, the characteristic constant in universal scaling function ($F_{+}$) is used to defined the global payoff matrix on the 2D grid. The mapping between *G* and *g* on the 2D grid is carried out numerically (shown in Fig. 4). The red line ($G=g$) represents the trivial relation for the fully-connected network. All data sit in the stable regime (blue background) and confirm that the dynamics on the 2D grid is always ecologically stable with $G>1$. The mapping between the local and the global gains in the 2D grid reveals strong renormalization due to the spatial structure of the network. The global gain provides a convenient method to compare the effects of population structures on the extinction time.

The global gain *G* turns out to be the appropriate rescaled variable. When plotted with the global gain, the extinction time on the 2D grid collapse onto the same scaling function $F_+(x)$ as shown in Fig. 5. Because the instability of the ecosystem is wiped out by the grid structure, the other branch $F_-(x)$ does not show up. The reappearance of universal scaling indicates that the population structure only renormalizes the phase boundary but the underlying extinction behavior remains the same.

**Figure 6:**
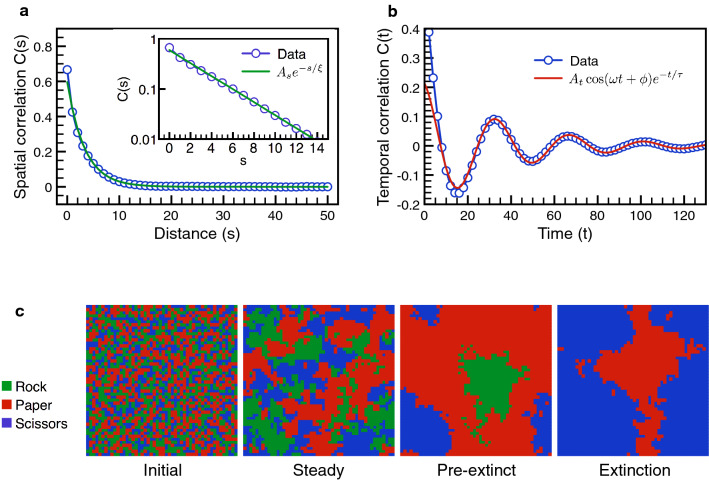
Correlation functions and the snapshots. The correlation function on the 2D grid is computed at $g=1$ with a large population size $N=10,000$ to reduce the unnecessary statistical errors. (**a**) The spatial correlation function describes the population distribution in space and signals the tendency for pattern formation. The numerical data is well fitted by the standard exponential decay, $A_{s}e^{-s/\xi }$ with the amplitude $A_{s}=0.596$ and correlation length $\xi =3.344$. Short correlation length and no oscillation in the spatial correlation indicate no pattern formation, agreeing with incoherent spiral wavelets observed in numerical simulations. (**b**) The temporal correlation is captured by $A_{t}\cos (\omega t+\phi )e^{-t/\tau }$ (red curve) with amplitude $A_{t}=0.218$, oscillatory frequency $\omega =0.183$, phase shift $\phi =0.215$ and correlation time $\tau =37.75$. The oscillation is due to competitions between clusters of different species, showing alternating dominance of each species in temporal sequence. (**c**) The snapshots of the ecological system at four different stages: initial, steady, pre-extinct and extinct states. In the initial state, the populations of all species are randomly distributed. After a short time (compare with the extinction time $T_{ex}$), the population profile enters the steady phase, characterized by emergence and demise of incoherent spiral wavelets. The ecosystem spends most of its lifetime in the steady phase before entering the pre-extinct state where a spiral wave comparable to the system size happens to take over and threatens the biodiversity. The pre-extinct state only lasts for a short period of time. Then, extinction sets in with one species eliminated completely.

### Spatial and temporal correlations on the grid

 To explore the different dynamics arisen from the network structure, it is helpful to study the spatial and temporal correlation functions,

19 $$\begin{aligned} C(r,t) \equiv \sum _{X}\langle X(r,t)X(0,0) \rangle -\langle X(r,t) \rangle \langle X(0,0) \rangle , \end{aligned}$$

where the summation is over all species, $X = A, B, C$. The random variable $X(r,t)=1$ when the species *X* is spotted at the distance *r* and in the later time *t*. Otherwise, its value is zero. The ensemble average $\langle X(r,t)X(0,0) \rangle$ measures the probability of finding the same species with the space-time separation (*r*, *t*) and equals $\langle X(r,t) \rangle \langle X(0,0) \rangle$ if no correlation is present. The cross-species correlation functions are neglected here because they reflect the same trend as observed in *C*(*r*, *t*).

The spatial and temporal dependences of the correlation function is shown in Fig. 6. The spatial correlation is short-ranged with correlation length $\xi \approx 3.3$ (lattice unit) for $g=1$. The data fits the usual exponential decay rather well and implies no long-ranged pattern formation in the 2D grid. The temporal correlation is more subtle – it shows non-trivial oscillations $\omega \approx 0.18$ with a relatively long correlation time $\tau \approx 37.8$. There exists clear oscillatory signature before the temporal correlation damps out. This oscillation in the steady state is not predicted in the replicative dynamics. Because the initial configuration is randomly sampled, the population size of each species is the same at the beginning. According to the replicator equations, the initial condition sits right on the fixed point and the dynamics is trivially static – no oscillation at all. One may cautiously argue that, due to statistical fluctuations, the initial condition is not exactly on the fixed point. Straightforward analysis leads to an intrinsic oscillation frequency $\omega _{0}=(g+1)/2\sqrt{3}$ near the fixed point. The sensitive dependence on the local gain *g* is not found in the numerics. Thus, the temporal oscillation must come from a different origin.

The situation is close to the nematic phase^73^ where the correlation in one direction (temporal) is stronger than the other (spatial). One can picture the whole system is roughly divided into many spiraling units of linear size $\xi$. Each unit generates local spiral waves but never synchronizes into the global spiral due to interactions with the wavefronts of other neighboring units. Because the spiral unit is local, its oscillation cannot be captured by the usual replicative dynamics which works for uniformly mixed populations. This picture purveys a simple explanation why the biodiversity is greatly enhanced in the 2D grid. Suppose the probability for a spiral unit to go out is *p*. The interactions among the units ignite or suppress the neighboring ones. Thus, the probability for the complete extinction $P_{ex}$ requires all units to go out at the same time, $P_{ex} \approx p^{N/\xi ^2}$, where $N/\xi ^2$ is roughly the number of spiraling units. The extinction is proportional to the reciprocal of $P_{ex}$,

20 $$\begin{aligned} T_{ex} \sim \frac{1}{P_{ex}} \approx p^{-N/\xi ^2} \quad \rightarrow \quad T_{ex} \sim e^{N/N_+}, \end{aligned}$$

where $N_+ = \xi ^2 / |\ln p|$. The exponential trend of the extinction time arises from the concurrent decease of the spiraling units. Although complete understanding of the evolutionary dynamics on the 2D grid is not yet achieved, the snap shots in Fig. 6 supports the qualitative picture proposed here.

## Discussion

Two major results are found in the agent-based Monte Carlo simulations. The first one is the emergence of universal scaling. The second one is the renormalized global payoff due to spatial structure of the network. While our numerical results clearly support the above conclusions, a rigorous proof remain open at the point of writing. In fact, some other numerical study^74^ on the cooperation in social games also found that the rescaled extinction time follows universal scaling. However, their emphasis is in the neutral regime (critical phenomena) where the power law reigns. But, in the rock-paper-scissors game we studied here, we find the phase transition can be collapsed onto two universal curves when plotted in terms of the rescaled variables. This is quite unexpected and shows that the universal scaling works not just in the neutral regimes (or equivalently, the criticality). Thus, our conclusions are stronger and can serve as an important method to classify different dynamical trends for ecosystems with finite populations.

One can try to understand the extinction trends qualitatively by the dynamics of the biodiversity indicator $\chi (t) \equiv a(t) b(t) c(t)$. When the population is infinite and well-mixed, its dynamics can be derived from the replicator equation,

21 $$\begin{aligned} \frac{d\chi }{dt} = (g-1) \Gamma (a,b,c) \chi , \end{aligned}$$

where $\Gamma (a,b,c)=1-3(ab+bc+ca) >0$ due to the constraint $a+b+c=1$ for the positive-definite frequencies. It is clear that the drift flows toward extinction ($\chi =0$) for $g<1$ while the ecosystem is stable for $g>1$. At the critical value $g=1$, the drift disappears and the flow is neutral.

Now consider the effect of finite population. At the critical point $g=1$, the biodiversity indicator is no longer a constant of motion but suffers an $\mathcal {O}(1/N)$ drift toward extinction^34^. The exponential trend for $g>1$ can be understood as a chain of successive rare events against the flow while the logarithmic trend for $g<1$ is the direct consequence of the exponential decay due to the drift toward extinction. Right at the critical point $g=1$, the neutral drift implies the absence of any characteristic time scale and thus hints for the emergence of the universal scaling. Detail calculations for the fluctuation-induced drift leads to the extinction time $T_{ex} \sim N$ and explains the exponent $\alpha =1$. However, it remains an open question why the classification of the dynamics can be readily described by the scaling arguments developed for phase transitions in thermostatistics.

The 2D grid posts even more challenging puzzles. The success of applying global gain *G* to collapse all data onto the same scaling function is encouraging for further studies along the route of renormalization-group techniques. This shall provides an independent check on the relation between *G* and *g*, which is obtained numerically in our study here. The plausible coarse-graining in the time domain is further supported from the prolonged correlation time in the temporal correlation. The renormalization group may also provide more information about the exponents and the scaling functions.

## Data Availability

The datasets used and/or analysed during the current study available from the corresponding author on reasonable request.
